# Recommendations from the AML molecular MRD expert advisory board

**DOI:** 10.1038/s41375-024-02275-x

**Published:** 2024-05-23

**Authors:** Stuart Scott, Alison Devonshire, Richard Dillon, Christian Thiede, Nicholas C. P. Cross, Helen E. White, Leandro Lo Cascio, Katya Mokretar, Nicola Potter, Christopher S. Hourigan, Jerald Radich, Adam Corner, Véronique Laloux, Gemma Halliday, Daniel Dilks, Tom Morrison, Katelyn Gilmour, Ashley Cartwright, Liam Whitby

**Affiliations:** 1 UK NEQAS for Leucocyte Immunophenotyping, Sheffield Teaching Hospitals NHS Foundation Trust, Sheffield, UK; 2 Department of Oncology and Metabolism, University of Sheffield, Sheffield, UK; 3 Molecular and Cell Biology, National Measurement Laboratory, LGC, Teddington, UK; 4 Department of Haematology, Guy’s International Centre of Excellence in Myeloid Disorders, Guy’s and St Thomas NHS Foundation Trust, London, UK; 5 Department of Medical and Molecular Genetics, King’s College, London, UK; 6 University Hospital Carl Gustav Carus, Dresden University of Technology, Dresden, Germany; 7 AgenDix, Applied Molecular Diagnostics, GmbH, Dresden, Germany; 8 Faculty of Medicine, University of Southampton, Southampton, UK; 9 Wessex Genomics Laboratory Service, Salisbury District Hospital, Salisbury, UK; 10 Diagnostics R&D, Medicines and Healthcare Products Regulatory Agency (MHRA), Potters Bar, UK; 11 Cancer Genetics, Guy’s Hospital, Synnovis, London, UK; 12 Laboratory of Myeloid Malignancies, National Heart, Lung, and Blood Institute, Bethesda, MD USA; 13 Translational Science & Therapeutics Division, Fred Hutchinson Cancer Center, Seattle, WA USA; 14 Digital Biology Group, Bio-Rad Laboratories, Pleasanton, CA USA; 15 Translational Science and Precision Diagnostics, QIAGEN, Courtaboeuf, France; 16 Horizon Discovery, Diagnostic Reference Standards, Cambridge, UK; 17 AccuGenomics, Inc., Wilmington, NC USA; 18 Clinical NGS and Oncology Division, Thermo Fisher, Paisley, UK

**Keywords:** Acute myeloid leukaemia, Cancer genomics

## To the Editor:

The standard-of-care treatment for patients with acute myeloid leukemia (AML) is being challenged by new classes of targeted therapies, including FLT3, IDH and BCL2 inhibitors [1]. Contemporaneously, measurable residual disease (MRD) testing is being considered as a surrogate for traditional clinical outcomes in the clinical trials of these new therapies [2]. To successfully integrate MRD assays into clinical trials, testing must be standardized between laboratories to ensure results are comparable in multicentre MRD assessment studies. Thus, results from different clinical trials can be meaningfully compared and, ultimately, clinical thresholds around common MRD levels applicable to all AML patients globally can be developed for these new treatments.

**Fig. 1:**
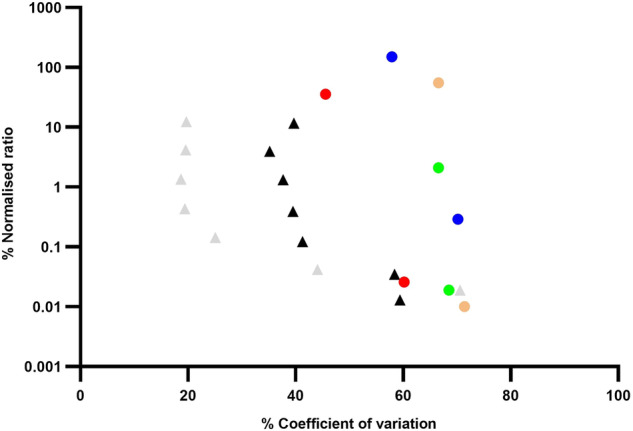
Comparison of interlaboratory variation for standardized *BCR*::*ABL1*^IS^ RT-dPCR (gray triangle markers) and RT-qPCR (black triangle markers) [5] testing with unstandardized *RUNX1*::*RUNX1T1* (blue circle markers), *CBFB*::*MYH11* (red circle markers), *PML*::*RARA* (green circle markers) and *NPM1* (orange circle markers) RT-qPCR testing [6].

To this end, the European LeukemiaNet (ELN) group published recommendations in 2018 [3], updated in 2021, that standardized the technical and reporting aspects of AML MRD testing for reverse transcription PCR (RT-PCR) and flow cytometry based approaches, with the 2021 update extending this to next generation sequencing (NGS) analysis and the use of MRD as a surrogate marker in clinical trials. Further to this, external quality assessment (EQA) programs have been established to assess the quality of testing [4], For molecular MRD testing, EQA data has shown that interlaboratory variation for molecular AML MRD testing was substantially greater than that seen for *BCR*::*ABL1* testing in chronic myeloid leukemia (CML), despite both tests being reverse transcription quantitative PCR-based (Fig. 1) [5, 6]. However, *BCR*::*ABL1* MRD testing has been subject to many years of standardization, including the development of the *BCR*::*ABL1* international scale (IS) [7].

An expert advisory board was established through the NHS Chief Scientific Officer’s Knowledge Transfer Partnership program to assess how molecular AML MRD testing could be further standardized [8]. This letter represents the findings of the board. The advisory board was composed of experts, including clinical scientists working in the establishment of UK AML MRD testing and those leading the standardization of *BCR*::*ABL1* testing in CML, clinicians experienced in managing patients with AML-based on MRD testing results, measurement science (metrology) experts and reference material producers. Representatives from commercial providers involved in manufacturing in vitro diagnostic (IVD) kits, reference standards and instrumentations were also present. Finally, representatives were present from the ELN David group. The Foundation for the National Institute of Health (FNIH) Biomarkers Consortium were consulted outside of the meeting. ELN David and the FNIH Biomarkers Consortium are both active in AML MRD standardization and were approached to ensure that any standardization recommendations were complementary to the activities of these groups.

The stated aims of the expert advisory board were to answer the following questions:

1. Would further standardization of molecular AML MRD be beneficial?
2. What markers should be standardized?
3. How should the standardization of these markers be prioritized?
4. What would be the best approaches to standardizing these markers?

To expedite the workings of the board, several surveys were performed in advance of the meeting: one for the experts involved in the meeting, one for the laboratories currently performing molecular AML MRD testing and another for commercial providers of IVDs, standards and platforms (Supplementary Tables 1–6).

**Fig. 2:**
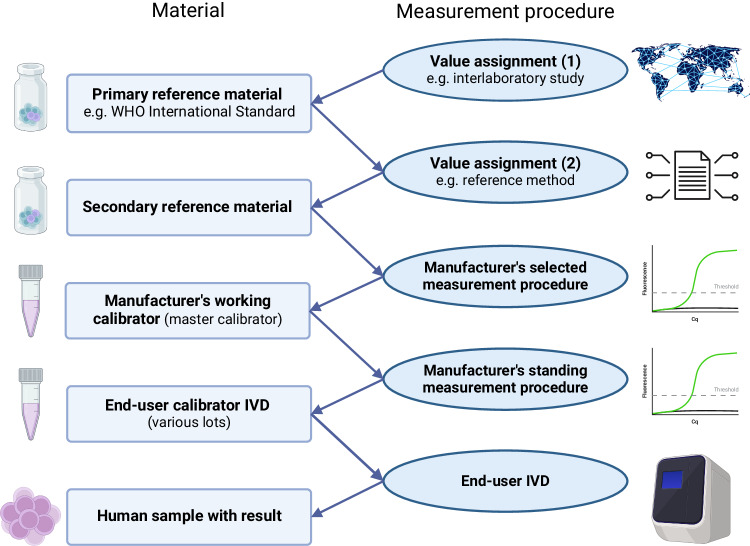
Structure of a calibration hierarchy whereby patient results are traceable to higher order reference materials such as World Health Organisation (WHO) International Standard materials, through a series of linked calibrations. An example of this is in the standardization of *BCR*::*ABL1*/reference gene ratio to the International Scale. Modified from ISO 17511:2020 Figure 4 model calibration hierarchy for “international conventional calibrator” that defines the quantity intended to be measured (“measurand”) [15]. IVD, in vitro diagnostic medical device. Created with BioRender.com.
- *Internationally recognized certification would be important to any reference materials produced, as is compliance with the European Union In Vitro Diagnostics Regulation (EU-IVDR)*. Certification of reference materials demonstrates that the material has been produced to a high standard, assuring laboratories of its quality, driving uptake. The EU-IVDR regulation, Chapter 1, Section 1, Article 1, point 3 specifically exempts certified reference materials (CRMs); however, they do require any IVD in-kit controls or calibrators to demonstrate traceability to reference materials of a higher metrological order; therefore, any reference materials produced should be of a sufficient order to satisfy this requirement and be available to IVD manufacturers for this purpose.
- The pre-board surveys suggested ISO 17034:2016 [11] or ISO 13485:2016 [12] would be the most appropriate form of certification for any reference materials produced (Supplementary Table 5); however, further discussion in the meeting suggested that *manufacturing reference materials according to World Health Organization (WHO) guidelines* [13] *and approval by the WHO Expert Committee on Biological Standardization (ECBS) would be the most appropriate approach given the current context of AML MRD testing where a reference method is not currently available.*
- *Higher order CRMs for RT-PCR based assays should be cell based* as this allows for full process control, capturing the uncertainty generated in the RNA extraction and cDNA synthesis processes known to impact on the final normalized ratio result (Supplementary Table 6).
- *Values for RT-PCR based MRD assays should only be assigned to any reference materials produced using ABL1 as a reference gene* to encourage standardization. Most laboratories performing molecular AML MRD testing use the *ABL1* reference gene to normalize testing results; however, a small subset uses alternative reference genes, such as *GUSB*, *B2M* and *HMBS*. The use of non-*ABL1* reference genes by around 15% of laboratories has proven problematic to the standardization of *BCR*::*ABL1* in CML, with conversion factors relating to *GUSB* being shown to be potentially more unstable when compared with their *ABL1* counterparts despite extensive standardization [14].
- *The development of research use only (RUO) or QC materials calibrated to the primary CRM would also be beneficial to the field*.

The recommendations of the board and pre-board survey have been outlined below:

- *All board members felt further standardization of molecular AML MRD testing would be beneficial to the field*.
- *The main blocks to standardization cited by EQA participants were lack of time and money* (Supplementary Table 1), therefore, any standardization efforts should be easy to implement and inexpensive (Supplementary Table 2).
- *Standardization of RUNX1::RUNX1T1, CBFB::MYH11 type A and NPM1 type A testing by RT-PCR-based approaches was deemed to be the priority* (Supplementary Table 3). These markers are some of the most prevalent somatic changes found in patients with AML. Furthermore, there are commercially available cell lines facilitating standardization projects. Despite the high prevalence of the *PML*::*RARA* rearrangement in AML patients, *PML*::*RARA* MRD testing is primarily interpreted qualitatively thus limiting the impact of any standardization projects [3].
- *RT-PCR-based testing of NPM1 type B and D and FLT3 internal tandem duplications (ITD) should be considered for future standardization projects*. *NPM1* type B and D are only found in around 10% and 8% of *NPM1* positive patients with AML, respectively, compared to 70% who have the *NPM1* type A variant [9]; however, this is still a substantial number of patients and standardization should be considered if stable cell lines can be produced. *Cell lines for NPM1 type B and D should be developed to facilitate future standardization projects*. Despite recent publications showing the prognostic importance of *FLT3* ITD MRD testing [10], it is not yet clear how this testing will be employed and what the clinically important cut-offs will be, thus it would be premature to begin standardizing testing.
- *The development of higher order, primary reference materials (RMs) was the priority of the board* (Supplementary Table 4). Primary RMs are at the top of the calibration hierarchy from which other quality control (QC) materials can be calibrated (Fig. 2).

These recommendations should act as a starting point for commercial and academic consortia to work together to develop the resources outlined above to ensure that clinical trials results, and ultimately the management of patients with AML, are both based on the highest quality data possible.

### Supplementary information

Supplemental Material
