# Supplementary material for: Recommendations from the AML molecular MRD expert advisory board
Source: Leukemia. 2024 May 23;38(7):1638–41. doi: 10.1038/s41375-024-02275-x (PMC11216975; doi:10.1038/s41375-024-02275-x)
Supplement: Supplementary file 1 — Supplemental Material [file 41375_2024_2275_MOESM1_ESM.docx]

**Supplemental data**

**Survey data**

Three surveys were performed in advance of the expert advisory board: one for the experts involved in the meeting, one for the laboratories currently performing molecular acute myeloid leukaemia (AML) measurable residual disease (MRD) testing (members of United Kingdon National External Quality Assessment Scheme for Leucocyte Immunophenotyping (UK NEQAS LI) external quality assessment programmes) and another of commercial providers of in vitro diagnostics, standards and platforms.

A total of 57 laboratories, from 20 countries responded to the laboratory survey: United Kingdom (n = 12), Germany (n = 8), Italy (n = 5), Switzerland (n = 4), Belgium (n = 4), Australia (n = 4), Spain (n = 3), Republic of South Africa (n = 2), Sweden (n = 2), Austria (n = 2), Singapore (n = 2), Croatia (n = 1), Finland (n = 1), Malaysia (n = 1), Norway (n = 1), Portugal (n = 1), Slovenia (n = 1), Czech Republic (n = 1), Netherlands (n = 1), China (n = 1).

Eight individuals responded to the expert survey. Five commercial providers responded to the commercial survey.

- 44/57 (77%) laboratories were interested in standardising their molecular AML MRD assays.
- 40/57 (70%) laboratories were interested in collaborating on any standardisation efforts.

**Supplementary Table 1. Blocks to standardisation reported by laboratories currently performing AML MRD testing (n = 49). Data from laboratories survey. Respondents could choose multiple relevant options.**

| **Blocks to standardisation** | **n** |
| --- | --- |
| Lack of time | 39 |
| Financial | 21 |
| Lack of understanding/training opportunities | 7 |
| Lack of staffing | 2 |

**Supplementary Table 2. Budget laboratories would be willing to spend per annum to standardise a single molecular AML MRD assay (n = 37).**

| **Budget** | **n** |
| --- | --- |
| >€5000 | 2 |
| €1000–5000 | 5 |
| €500–1000 | 9 |
| €200–500 | 11 |
| <€200 | 10 |

**Supplementary Table 3. Priority markers for standardisation. Respondents were asked to rank the priority levels on a scale from 1 (high) to 5 (low). The outcomes in the tables represent the average ranking of each of the subgroups surveyed.**

| **Marker** | **Priority (high = 1; low = 5)** | | |
| --- | --- | --- | --- |
|  | **Experts  (n = 7)** | **Laboratories  (n = 39)** | **Commercial**  **(n = 3)** |
| *RUNX1*::*RUNX1T1* | 1.0 | 2.2 | 1.0 |
| *NPM1* type A | 1.1 | 2.3 | 1.0 |
| *NPM1* type B & D | 1.6 | 3.1 | 1.3 |
| *CBFB*::*MYH11* type A | 1.7 | 2.2 | 1.0 |
| *FLT3* ITD | 1.8 | 2.4 | 1.0 |
| *NPM1* rare variants (>40) | 2.0 | 3.1 | 1.0* |
| *PML*::*RARA* bcr2 | 2.0 | 2.9 | 3.5 |
| *PML*::*RARA* bcr1 | 2.1 | 2.6 | 2.0 |
| *PML*::*RARA* bcr3 | 2.1 | 2.8 | 3.5 |
| *CBFB*::*MYH11* type D | 2.3 | 3.4 | 3.0 |
| *CBFB*::*MYH11* type E | 2.3 | 3.5 | 3.0 |
| *CBFB*::*MYH11* other | 4.4 | 3.3 | n/a |

**Supplementary Table 4. Preferred standardisation approach. Respondents were asked to rank the priority levels on a scale from 1 (high) to 5 (low). The outcomes in the tables represent the average ranking of each of the subgroups surveyed.**

| **Standardisation approach** | **Priority (high = 1; low = 5)** | | |
| --- | --- | --- | --- |
|  | **Experts  (n = 8)** | **Laboratories (n = 33)** | **Commercial (n = 5)** |
| Development of a primary certified reference material | 1.8 | 2.3 | 1.6 |
| Development of research use only (RUO) calibration or quality control materials | 1.9 | 2.6 | 1.8 |
| Development and validation of a reference measurement procedure | 2.4 | 2.7 | 1.8 |
| Development of a sample exchange programme to allow conversion of local results to a common scale | 3.0 | 2.9 | 3.7 |

**Supplementary Table 5. Preferred certification for reference material. Respondents were asked to rank the priority levels on a scale from 1 (high) to 5 (low). The outcomes in the tables represent the average ranking of each of the subgroups surveyed.**

| **Certification** | **Priority (high = 1; low = 5)** | | |
| --- | --- | --- | --- |
|  | **Experts  (n = 6)** | **Laboratories (n = 30)** | **Commercial (n = 3)** |
| Certified reference material (general requirements for the competence of reference material producers eg European Reference Material; produced under international organisation for standardization (ISO) 17034 accreditation) | 1.8 | 2.3 | 2.0 |
| Reference standards produced under ISO 13485 (medical devices – quality management systems) accreditation | 1.8 | 2.5 | 2.3 |
| World Health Organisation established International Standard | 2.0 | 2.6 | 2.0 |
| Conformité Européene (CE) in vitro diagnostic (IVD) marking | 2.0 | 2.9 | 2.0 |
| UK Conformity Assessed IVD Marking [https://www.gov.uk/guidance/regulating-medical-devices-in-the-uk#UKCA] | 2.2 | 3.2 | 2.3 |
| RUO (with Certificate of Analysis available/produced under current good manufacturing practice) | 2.5 | 3.0 | 2.7 |
| United States Food and Drug Administration approval | 3.5 | 3.4 | 1.7 |

**Supplementary Table 6. Preferred reference material matrix. Respondents were asked to rank the priority levels on a scale from 1 (high) to 5 (low). The outcomes in the tables represent the average ranking of each of the subgroups surveyed.**

| **Reference material** | **Priority (high = 1; low = 5)** | | |
| --- | --- | --- | --- |
|  | **Experts  (n = 8)** | **Laboratories  (n = 31)** | **Commercial**  **(n = 2)** |
| Cell based | 1.1 | 2.8 | 3.5 |
| DNA (eg plasmid) | 3.0 | 2.7 | 1.0 |
| RNA (eg purified cell line RNA and/or *in vitro* transcribed RNA) | 3.0 | 2.7 | 1.0 |
| Armoured RNA | 3.8 | 2.9 | 4.5 |
